# Myocardial contrast echocardiography assessment of perfusion abnormalities in hypertrophic cardiomyopathy

**DOI:** 10.1186/s12947-022-00293-2

**Published:** 2022-09-19

**Authors:** Paola Roldan, Sriram Ravi, James Hodovan, J. Todd Belcik, Stephen B. Heitner, Ahmad Masri, Jonathan R. Lindner

**Affiliations:** 1 Knight Cardiovascular Institute, Oregon Health and Science University, Portland, OR USA; 2 Division of Cardiovascular Medicine, University of Virginia Medical Center, 415 Lane Rd, CVRC Box 801394, Charlottesville, VA 22908 USA

**Keywords:** Hypertrophic cardiomyopathy, Ischemia, Myocardial contrast echocardiography

## Abstract

**Background:**

Perfusion defects during stress can occur in hypertrophic cardiomyopathy (HCM) from either structural or functional abnormalities of the coronary microcirculation. In this study, vasodilator stress myocardial contrast echocardiography (MCE) was used to quantify and spatially characterize hyperemic myocardial blood flow (MBF) deficits in HCM.

**Methods:**

Regadenoson stress MCE was performed in patients with septal-variant HCM (*n* = 17) and healthy control subjects (*n* = 15). The presence and spatial distribution (transmural diffuse, patchy, subendocardial) of perfusion defects was determined by semiquantitative analysis. Kinetic analysis of time-intensity data was used to quantify MBF, microvascular flux rate (β), and microvascular blood volume. In patients undergoing septal myectomy (*n* = 3), MCE was repeated > 1 years after surgery.

**Results:**

In HCM subjects, perfusion defects during stress occurred in the septum in 80%, and in non-hypertrophied regions in 40%. The majority of septal defects (83%) were patchy or subendocardial, while 67% of non-hypertrophied defects were transmural and diffuse. On quantitative analysis, hyperemic MBF was approximately 50% lower (*p* < 0.001) in the hypertrophied and non-hypertrophied regions of those with HCM compared to controls, largely based on an inability to augment β, although hypertrophic regions also had blood volume deficits. There was no correlation between hyperemic MBF and either percent fibrosis on magnetic resonance imaging or outflow gradient, yet those with higher degrees of fibrosis (≥ 5%) or severe gradients all had low septal MBF during regadenoson. Substantial improvement in hyperemic MBF was observed in two of the three subjects undergoing myectomy, both of whom had severe pre-surgical outflow gradients at rest.

**Conclusion:**

Perfusion defects on vasodilator MCE are common in HCM, particularly in those with extensive fibrosis, but have a different spatial pattern for the hypertrophied and non-hypertrophied segments, likely reflecting different contributions of functional and structural abnormalities. Improvement in hyperemic perfusion is possible in those undergoing septal myectomy to relieve obstruction.

**Trial registration:**

ClinicalTrials.gov NCT02560467.

**Graphical Abstract:**

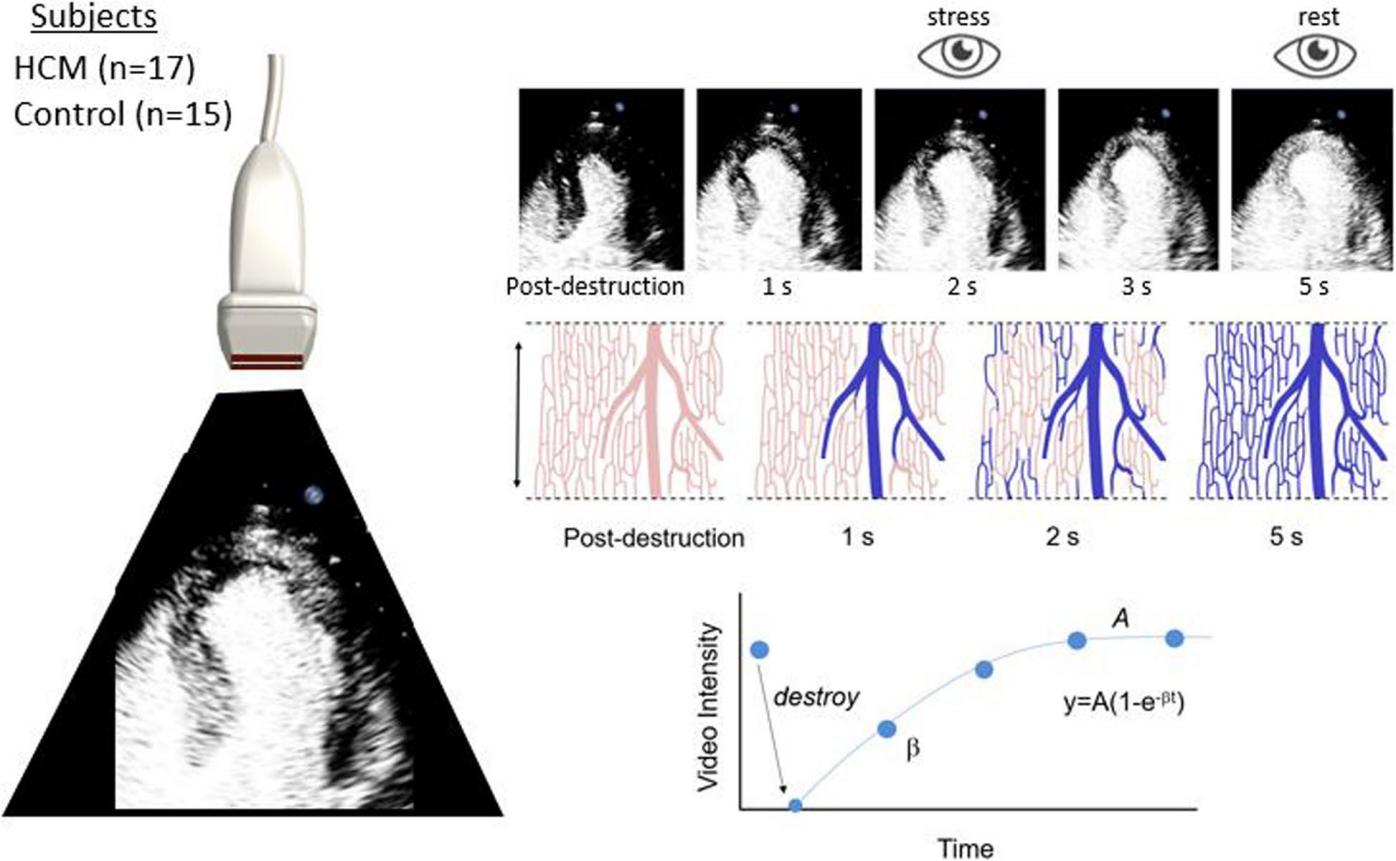

**Supplementary Information:**

The online version contains supplementary material available at 10.1186/s12947-022-00293-2.

Abnormalities in myocardial perfusion in the absence of coronary artery disease (CAD) occur in patients with hypertrophic cardiomyopathy (HCM) [1–4]. While fixed perfusion defects can occur from extensive HCM-related myocardial fibrosis [5], there is also a high prevalence of abnormal myocardial blood flow (MBF) reserve during vasodilator testing [1, 3]. In some studies, reduced flow reserve has been attributed to low hyperemic perfusion rather than from the high resting flow that can occur from hypertrophy-related increased wall stress and oxygen demand [1, 6]. The presence of a relationship between inducible ischemia and the degree of either myocardial fibrosis or left ventricular outflow tract gradients in HCM is uncertain based on mixed results [1, 3, 4, 7]. Less controversial is the association between inducible ischemia and adverse clinical outcomes [1, 3].

Ischemia in the absence of CAD in HCM has been attributed to structural abnormalities such as microvascular rarefaction and arteriolar medial hyperplasia [8–10]. Functional abnormalities are also possible from exaggerated systolic flow reversal and delay in diastolic forward flow in the distal coronary arteries, or from epicardial-endocardial perfusion pressure loss [11–13]. These mechanisms could contribute to ischemia in non-hypertrophied segments which has been reported in HCM and even in high-risk gene carriers [14, 15]. In the current study, vasodilator stress myocardial contrast echocardiography (MCE) perfusion imaging was performed in patients with septal-variant HCM to assess the prevalence of reversible ischemia in both the hypertrophied and non-hypertrophied regions; and to spatially characterize ischemia as transmural diffuse, patchy, or subendocardial in distribution. Using parametric analysis, abnormalities in MBF at rest or during vasodilator stress were classified as being attributable to an impairment in microvascular flux or from loss of functional microvascular units. In a small subset of subjects undergoing septal myectomy for symptomatic obstruction, repeat MCE was performed to assess for changes in myocardial MBF reserve based on the potential impact of improving vascular function by reducing late intraventricular systolic pressures.

## Methods

### Subjects

The study was approved by the Investigational Review Board at Oregon Health & Sciences University and registered with ClinicalTrials.gov (NCT02560467). The study design was a prospective, non-blinded study of seventeen subjects between the ages of 19 and 70 with a diagnosis of septal variant HCM and fifteen age-matched subjects free of cardiac symptoms with no more than one CAD risk factor (lipid disorder, hypertension, diabetes, smoking) who were recruited to serve as normal controls. Subjects with HCM were recruited if they had a diagnosis made by echocardiography or cardiac magnetic resonance imaging (CMR) with a maximal septal thickness of 15 mm or greater, and also had undergone CMR with late gadolinium enhancement (LGE) imaging for quantification of fibrosis within the preceding 6 months. Subjects were excluded for known coronary or peripheral artery disease, significant valvular heart disease other than that caused by systolic anterior motion which could be no more than moderate in severity, history of resuscitated sudden cardiac death (SCD), left ventricular (LV) systolic dysfunction (ejection fraction [LVEF] < 50%), pregnancy, contraindications to regadenoson, allergy to ultrasound enhancing agents, or elite athlete status. Additional exclusion criteria for HCM subjects included prior septal reduction therapy (either surgical or alcohol ablation), pacemaker-dependent rhythm, presence of LV aneurysm, or treatment with cardiac myosin inhibitor.

### Symptom status and SCD risk

Angina symptoms in subjects with HCM were determined by history. Risk for SCD was determined by an established risk prediction model based on subject age, echocardiographic indices, history of arrhythmia, symptoms, and family history [16].

### Vasodilator stress myocardial contrast echocardiography

Vasodilator stress MCE was performed in all subjects and was repeated at least 12 months after myectomy in those who were referred for surgical septal reduction. Subjects abstained from caffeine for 48 h. MCE perfusion imaging (iE33, Philips Ultrasound, Andover, MA) was performed at a centerline frequency of 2.0 MHz with multi-pulse amplitude-modulation imaging at a mechanical index of 0.12–0.16. Overall gain was adjusted to levels just under those that produced background myocardial speckle. Images were acquired in the apical 4-chamber, 2-chamber, and long-axis imaging planes. Lipid-shelled microbubbles with a gas core containing either sulfur hexafluoride (Lumason, Bracco Diagnostics, Monroe Township, NH) or octafluoropropane (Definity, Lantheus Medical Imaging, North Billerica, MA) were used. Lumason was reconstituted in 5 mL of normal saline while activated Definity was diluted to 30 mL total volume in normal saline. Infusion rates were kept constant for each individual at a rate of 1.0 to 1.5 ml/min. A 5-frame high-power (mechanical index > 0.9) sequence was applied to destroy microbubbles in the imaging sector through inertial cavitation, after which electrocardiographically-triggered end-systolic frames were acquired until visual replenishment had occurred. MCE was performed at rest and during vasodilator stress produced by intravenous administration of regadenoson (0.4 mg). Heart rate and blood pressure were recorded at baseline and three minutes after injection of regadenoson.

### MCE analysis

Analysis was performed by a reader blinded to MRI and clinical data, other than diagnosis of HCM which is readily apparent on the echocardiogram. Perfusion was qualitatively defined as abnormal if there was lack of complete microvascular refill within 5 s at rest, or within 2 s during vasodilator stress [17] (See Additional file 1) Perfusion abnormalities were categorized as being evenly transmural, subendocardial, or patchy in appearance. Quantitative perfusion analysis was performed using software developed for MCE perfusion imaging (iMCE, Narnar LLC, Portland, OR). For control subjects, data were averaged from transmural regions-of-interest placed over each perfusion territory of all three major coronary arteries. For HCM subjects, transmural regions-of-interest were separately drawn over the hypertrophic and non-hypertrophic control subjects in either the 4-chamber or 3-chamber view. Regions with obvious rib artifact or cavity attenuation were excluded. The first frame after inertial cavitation was digitally subtracted from all subsequent frames and background-subtracted time-intensity data were fit to the function:

$$\mathrm y=\mathrm A(1-\mathrm e^{-\mathrm{\beta}t})$$

where *y* is signal intensity at time *t*, *A* is the plateau intensity reflecting relative microvascular blood volume (MBV), and *β* is the rate constant reflecting microvascular blood flux rate. Myocardial MBF was quantified by the product of MBV and β [18].

### Echocardiography

Echocardiography (iE33, Philips Ultrasound, Andover, MA) was performed to assess chamber dimensions, wall thickness, left ventricular function, and peak LVOT gradient according to guidelines published by the American Society of Echocardiography [19]. LV volumes, LVEF, and stroke volume in HCM subjects were calculated using the modified Simpson’s method. Stroke volume in controls was calculated by the product of LVOT area and time-velocity integral measured by pulsed-wave spectral Doppler. LV stroke work index was calculated by:

$$0.0136\times\mathrm{mean}\,\mathrm{arterial}\,\mathrm{pressure}\times\mathrm{stroke}\,\mathrm{volume}\,\mathrm{index}$$

For HCM subjects, LVOT gradient was added to mean arterial pressure, although this approach overestimates actual work based on the end-systolic nature of the gradient. Myocardial work index was calculated by the product of stroke work index and heart rate. The LVOT gradient in subjects with HCM were measured both at rest and during vasodilator stress upon completion of MCE perfusion imaging.

### Assessment of fibrosis by CMR

A standardized CMR protocol was performed on a 1.5 Tesla (T) scanner (Philips 1.5 Achieva or Integra) with multi-channel channel phased-array chest coils and electrocardiographic gating. Cine steady-state free precession imaging was performed covering the whole heart in 8 mm thick slices, though these data were not used for analysis. For LGE, a phase-sensitive inversion-recovery sequence was acquired 12–15 min after intravenous gadolinium contrast administration (0.2 mmol/kg). Distribution and extent of LGE was assessed both visually and quantitatively using the six standard-deviation threshold according to the Society for Cardiovascular Magnetic Resonance standards [20].

### Statistical analysis

Data were analyzed using Prism (version 9.0, GraphPad, San Diego, CA). For data determined to be normally distributed by the D’Agostino and Pearson omnibus test, differences were assessed by one-way ANOVA with post-hoc comparisons made by paired or unpaired Student’s *t*-test with Tukey’s test to adjust for multiple comparisons. Unless otherwise described, normally-distributed data are expressed as mean ± standard deviation. Differences for non-normally distributed data were assessed with Friedman’s test with post-hoc individual comparisons by Mann–Whitney U test for non-paired data or Wilcoxon signed-rank test for paired data. Non-normally treated data are expressed as median with interquartile range (IQR). Differences in proportions were compared using χ^2^ analysis. Relationship between MCE perfusion data and other clinical data were determined using either Spearman’s rank correlation coefficient (ρ) or Pearson correlation coefficient. Differences were considered significant at *p* < 0.05.

## Results

### Clinical characteristics

Vasodilator stress MCE could be performed in all control subjects, although quantitative MCE analysis was deemed unreliable in two subjects because of poor image quality. Two HCM patients were excluded because of either severe symptoms with regadenoson requiring immediate reversal with aminophylline, or discovery of multivessel CAD on angiography after stress MCE and LGE revealed abnormalities in a pattern typical for CAD.

Clinical characteristics of the final study group are shown in Table 1. For subjects with HCM, 40% had a history of angina. The number of risk factors for CAD and use of cardiovascular medical therapy tended to be greater in the HCM group, although the only individual feature that was significantly different between groups was the use of disopyramide and beta blockers. Key echocardiography variables are shown in Table 2. As expected, septal end-diastolic wall thickness, LVEF, and LVOT pressure gradient at rest were greater in HCM compared with normal control subjects. There were non-significant trends for smaller cavity dimensions in HCM. LV stroke work index and myocardial work index, which were calculated inclusive of end-systolic LVOT pressure gradients, were significantly higher in HCM than control subjects.

**Table 1:** Clinical characteristics and medications

	Control Subjects	HCM Subjects
	(*n* = 15)	(*n* = 15)
Age (years)	48 ± 6	54 ± 12
Sex (Male/Female)	7/8	11/4
BMI (kg/m^2^)	29.3 ± 7.7	30.1 ± 5.2
Family history of HCM, n (%)	0 (0%)	5 (33%)
Diabetes mellitus, n (%)	1 (6%)	0 (0%)
Hypertension, n (%)	3 (20%)	5 (33%)
Hyperlipidemia, n (%)	3 (20%)	8 (53%)
Smoking history, n (%)	4 (26%)	1 (6%)
Ventricular tachycardia, n (%)	0 (0%)	2 (13%)
Angina, n (%)	0 (0%)	6 (40%)*
ICD (%)	0 (0%)	2 (13%)
HCM 5-year SCD risk (%)		2.9 ± 1.2
NT-proBNP, median (pg/mL) [IQR]		285 [91–910]
Gene mutation positive, n (%)		6 (47%)
Medications, n (%)		
ACE-I/ARB	4 (26%)	2 (13%)
Beta blocker	4 (26%)	9 (60%)
Non-DHP calcium channel blocker	1 (6%)	4 (26%)
Disopyramide	0 (0%)	5 (33%)*
Amiodarone	0 (0%)	1 (6.7%)
Anti-platelet	5 (33%)	4 (26%)
Warfarin	0 (0%)	1 (6.7%)

*ACE-I* Angiotensin converting enzyme inhibitor, *ARB* Angiotensin receptor blocker, *BMI* Body mass index, *DHP* Dihydropyridine, *HCM* Hypertrophic cardiomyopathy, *ICD* Implantable cardiac defibrillator, *IQR* Interquartile range, *NT-proBNP* N- terminal pro-hormone B-type natriuretic peptide, *SCD* Sudden cardiac death

^*^p < 0.05 vs controls

**Table 2:** Echocardiography measurements at rest

	Control Subjects	HCM Subjects
	(*n* = 15)	(*n* = 15)
LVIDd (cm)	4.7 ± 0.6	4.3 ± 0.6
LVIDs (cm)	3.2 ± 0.8	2.7 ± 0.5
LV end-diastolic volume (mL)	102 ± 25	72 ± 18*
LV end-systolic volume (mL)	38 ± 10	30 ± 10
IVSd (cm)	0.9 ± 0.3	1.9 ± 0.4*
PWd (cm)	1.0 ± 0.3	1.2 ± 0.4
LVEF (%)	63 ± 7	72 ± 8*
Stroke volume (mL)	71 ± 10	80 ± 25
Stroke volume index (mL/m^2^)	34 ± 11	38 ± 11
Cardiac output (L/min)	4.9 ± 0.7	4.9 ± 1.2
Resting LVOT gradient (mm Hg) [IQR]	4 [3–6]	26 [8–52]*
LV stroke work index (g/m)	42 ± 10	98 ± 65*
LV myocardial work index (g/m/min)^a^	2870 ± 855	6014 ± 3720*

*IVSd* Diastolic interventricular septum thickness, *LVEDD* Left ventricular end-diastolic volume, *LVEF* Left ventricular ejection fraction, *LVESD* Left ventricular end-systolic volume, *LVIDd* Diastolic left ventricular diameter, *LVIDs* Systolic left ventricular diameter, *PWd* Diastolic posterior wall thickness

^a^Load component in HCM subjects calculated by summing mean arterial pressure and peak LVOT gradient

^*^p < 0.05 versus control subjects

### Perfusion imaging

Vital signs and hemodynamic measurements at rest and during vasodilator stress are shown in Table 3. An increase in heart rate and rate-pressure product during regadenoson stress was seen in control subjects but not HCM subjects, probably as a result of more frequent use of beta blockers and calcium channel antagonists in the HCM cohort. There was no major change in peak LVOT gradient from rest to vasodilator stress stage in the HCM cohort.

**Table 3:** Vital signs and hemodynamic measurements at rest and during vasodilator stress

	Control Subjects		HCM Subjects	
	(*n* = 15)		(*n* = 15)	
	Rest	Stress	Rest	Stress
Heart rate, min^−1^	70 ± 13	86 ± 20*	63 ± 10	73 ± 13
Systolic BP, mm HG	116 ± 17	115 ± 16	138 ± 17	132 ± 16
Diastolic BP, mm Hg	68 ± 12	70 ± 15	75 ± 7	74 ± 9
Rate-pressure Product	8,203 ± 2198	10,026 ± 3698*	8,795 ± 2038	9,538 ± 1850
Peak LVOT gradient, mm Hg (IQR)	-	-	26 (8–52)	32 (9–60)

*BP* Blood pressure, *IQR* Interquartile range, *LVOT* Left ventricular outflow tract

^*^p < 0.05 for stress versus rest

**Fig. 1:**
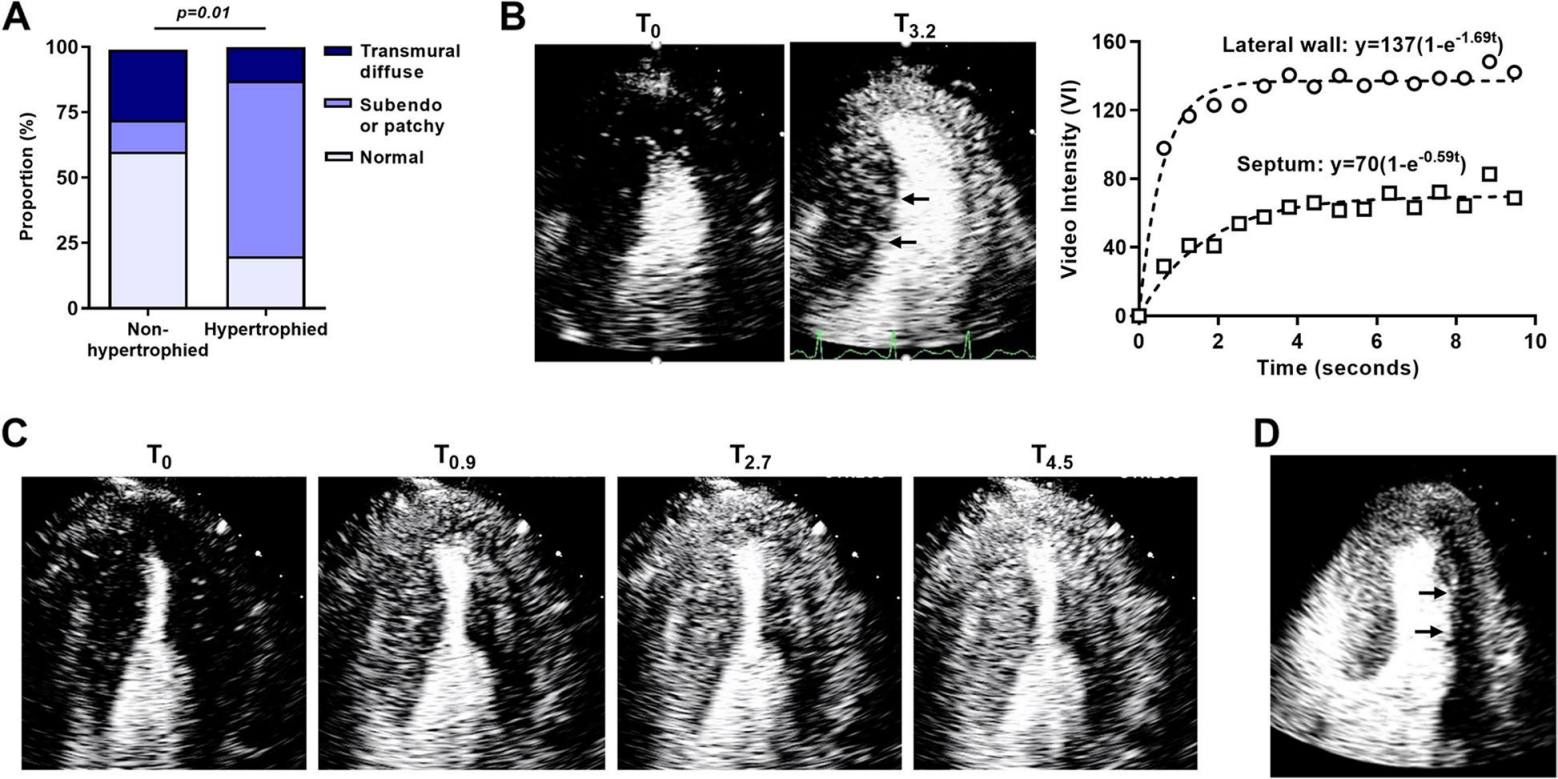
MCE detection of abnormal perfusion during vasodilator stress in HCM subjects. **A** Percentage of subjects with transmural diffuse, subendocardial or patchy, or normal perfusion during regadenoson for hypertrophied and non-hypertrophied control regions. **B** Example of end-systolic MCE images (apical 4-chamber) and corresponding time-intensity curves during vasodilator stress illustrating a patchy perfusion defect of the hypertrophied septum (arrows) and both reduced microvascular flux rate (β) and MBV (A-value). Images are shown immediately after microbubble destruction (T_0_) and at 3.2 s after refill (T_3.2_). **C** Vasodilator MCE (4-chamber view) illustrating delayed refill in all myocardial segments that was worse in the subendocardium. **D** Vasodilator MCE (4-chamber view) > 5 s after refill illustrating a transmural severe defect of the non-hypertrophied lateral wall (arrows)

On qualitative analysis of MCE, myocardial perfusion was normal at rest and during vasodilator stress in all but one of the control subjects who had mild global diffuse reduction contrast replenishment. In two HCM subjects (13%), patchy or diffuse hypoperfusion of the hypertrophied septum was observed at rest. During vasodilator stress, perfusion defects were observed in the majority of HCM subjects (Fig. 1). These defects were more common in the hypertrophied than non-hypertrophied segments. The spatial distribution of these perfusion defects was most commonly subendocardial or patchy rather than transmural and diffuse (Fig. 1). Defects in the non-hypertrophied territories were seen in over one-third of subjects in which case they tended to be transmural and diffuse.

**Fig. 2:**
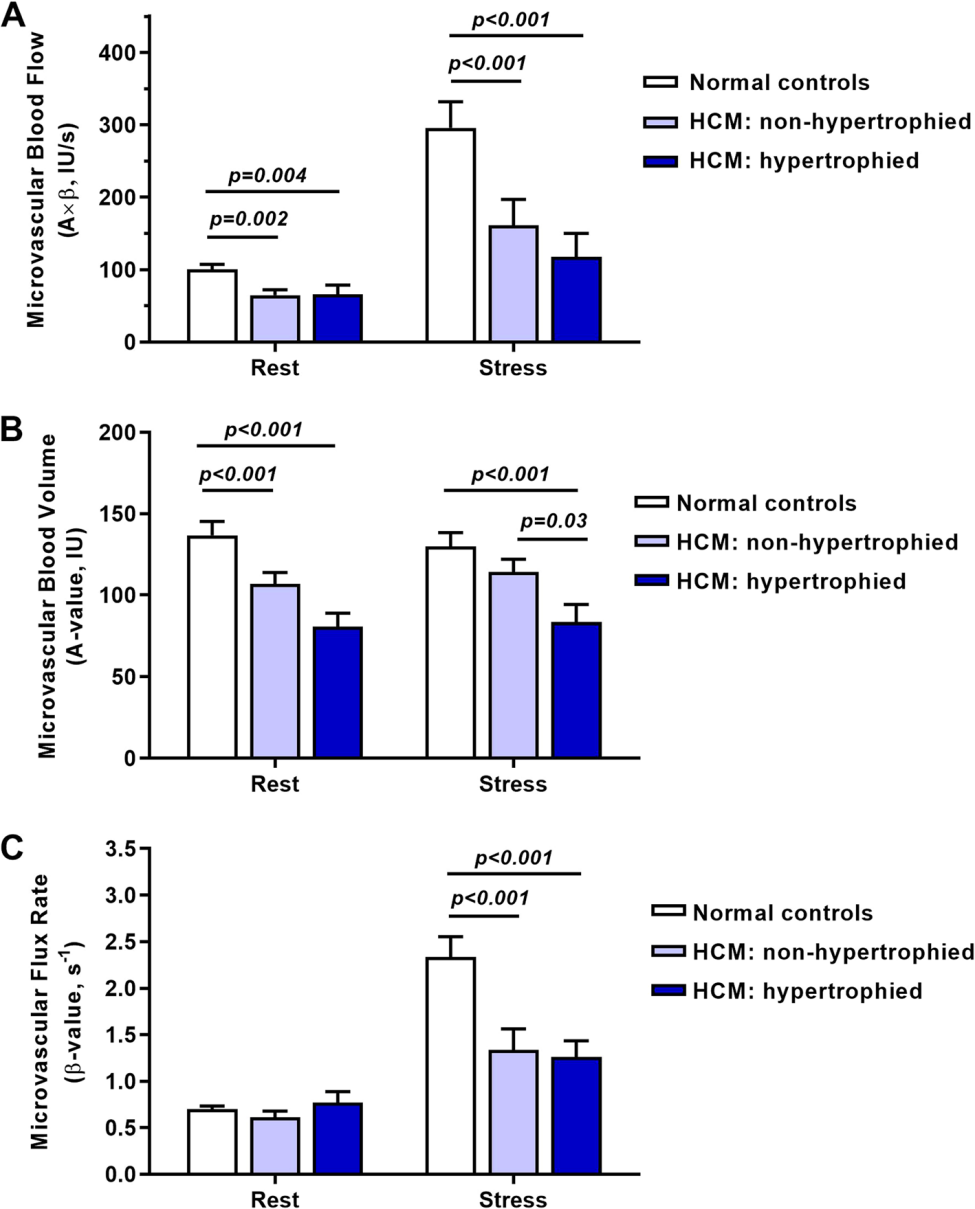
Quantitative MCE perfusion data (mean ± SEM) at rest and during vasodilator stress from normal control subjects and in the non-hypertrophied and hypertrophied regions from HCM subjects. Data include: (**A**) microvascular blood flow, (**B**) microvascular blood volume, and (**C**) Microvascular flux rate (β) derived from time-intensity data

On quantitative analysis (Fig. 2), MBF in both the hypertrophied and non-hypertrophied segments in HCM patients was modestly lower than in normal control subjects despite echocardiographically-determined myocardial work being higher in those with HCM. Consistent with previous studies, regadenoson increased MBF primarily through an increase in microvascular flux rate (β) [21]. During vasodilator stress, differences in MBF between HCM and normal control subjects were greater than at rest, which was attributable primarily to slower microvascular flux rate, although MBV in hypertrophied segments in HCM was persistently lower than in non-hypertrophied segments or in control subjects.

### Perfusion abnormalities and HCM clinical variables

**Fig. 3:**
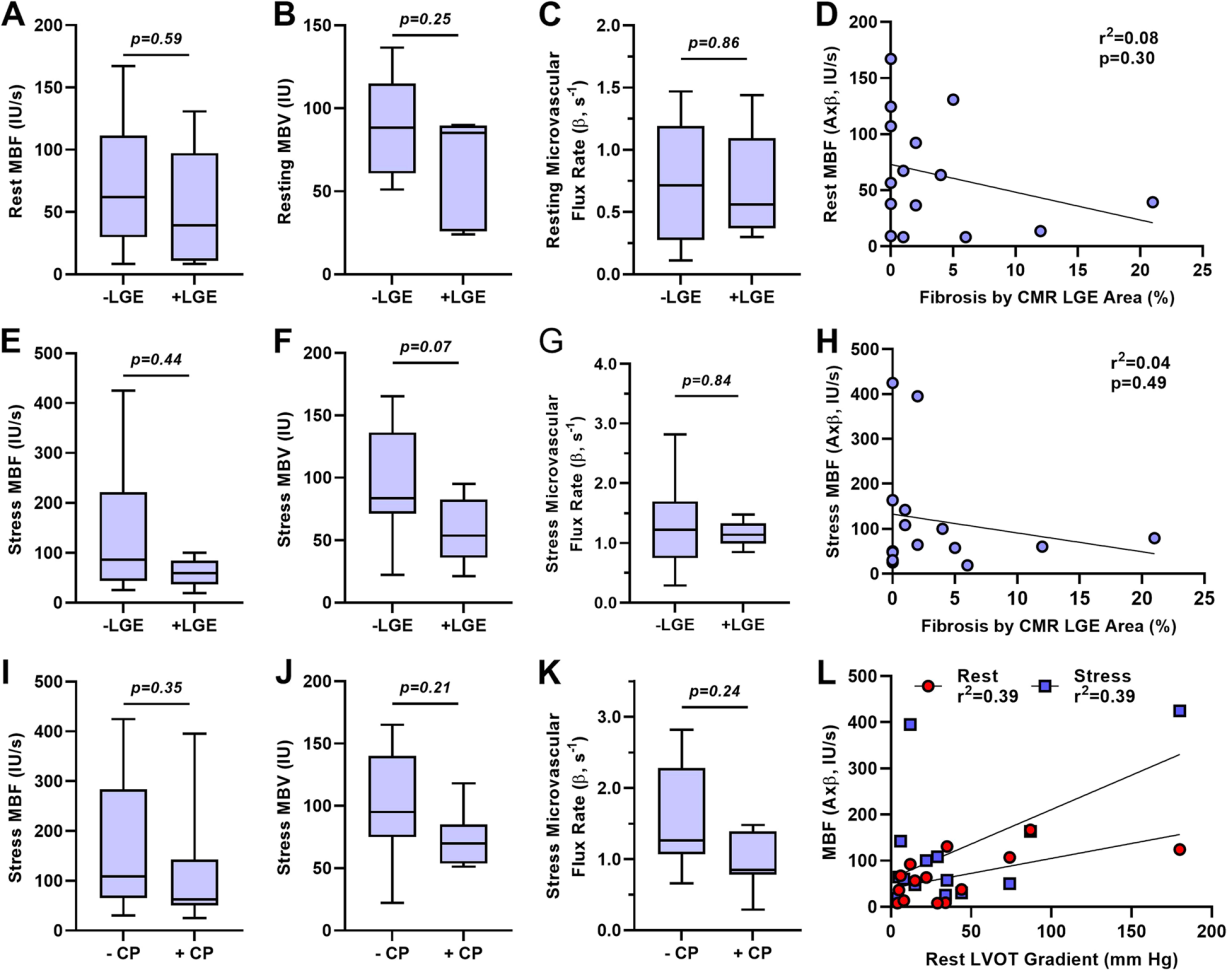
Quantitative MCE at rest (**A-C**) and during vasodilator stress (**E–G**) for microvascular blood flow (*MBF*), microvascular blood volume (*MBV*) and microvascular flux rate according to presence or absence of fibrosis > 2% by late gadolinium enhancement (LGE) on cardiac magnetic resonance imaging (*CMR*). (**D** and **H**) Relation between percent fibrosis by LGE and MBF at rest or stress. (**I-K**) Quantitative MCE during vasodilator stress according to the presence or absence of a history of anginal chest pain (*CP*). (**E–G**) Relation between left ventricular outflow tract (*LVOT*) gradient at rest and MBF at rest or during vasodilator stress

On CMR, the median LV fibrosis expressed as a percent of LV area with LGE was 2.0% (95% CI: 0.0–6.0). The presence of myocardial fibrosis defined as > 2% myocardial area was present in six subjects and was found only in hypertrophied segments. All six subjects with fibrosis were graded as having abnormal vasodilator stress MCE perfusion imaging. Quantitative MCE at rest and during stress in the hypertrophic segment was not significantly different according fibrosis status, and there was no relationship between fibrosis area and microvascular perfusion (Fig. 3A to H). Yet, subjects with significant fibrosis, defined as > 5%, all tended to have low MBF at rest and during stress. For subjects with a history of anginal chest pain (*n* = 6), obstructive CAD had been excluded by angiography in half. Myocardial perfusion and parameters of microvascular flux rate and MBV during stress in the hypertrophic segment was not significantly different according to anginal status (Fig. 3I to K). There was no significant relationship between LVOT gradient at rest and myocardial perfusion at rest or during stress (Fig. 3L). There was also no significant relationship between septal thickness and myocardial perfusion at rest or during stress (*p* = 0.98).

### Changes in myocardial perfusion after myectomy

**Fig. 4:**
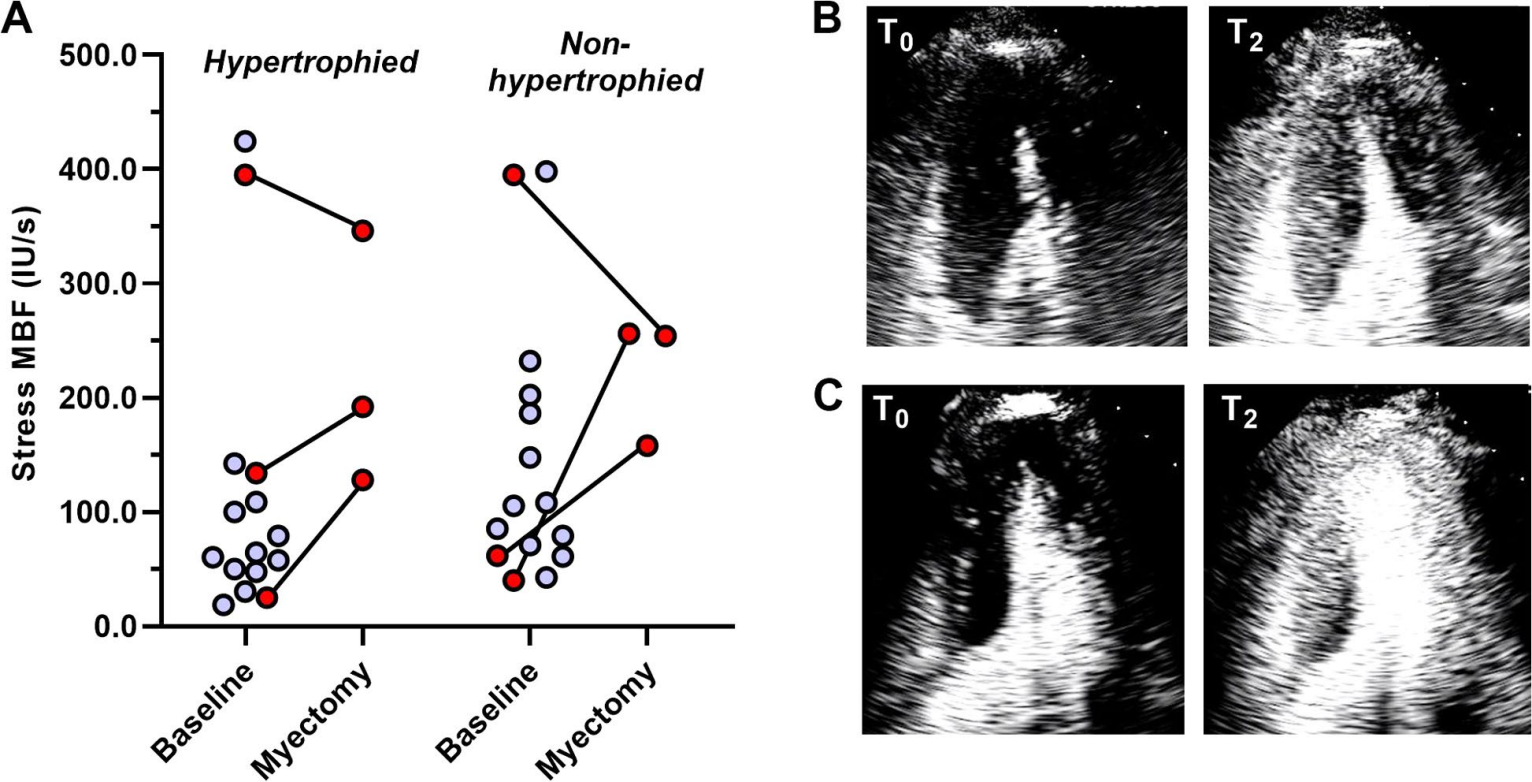
**A** Myocardial blood flow (*MBF*) in the hypertrophic and non-hypertrophic segments in patients with HCM during vasodilator stress showing individual data from the initial study (*baseline*), and after surgical myectomy (*n* = 3 subjects, data points in red). End-systolic images in the apical 4-chamber view during vasodilator MCE from a single for the study prior to myectomy **B** and more than one year after myectomy **C**. End-systolic images shown were acquired immediately after microbubble destruction (*T*_*0*_) and at approximately two seconds (*T*_*2*_) after replenishment and illustrate improvement in contrast enhancement in the hypertrophied and remote segments

Three subjects with HCM underwent surgical myectomy with or without papillary muscle realignment, two of whom had severe resting LVOT obstruction (> 100 mm Hg gradient), and one had severe obstruction (80 mm Hg) only during low intensity exercise. The post-myectomy LVOT gradient at rest was < 15 mm Hg in all three subjects, and the peak gradient during exercise was 23 to 52 mm Hg. Repeat vasodilator stress MCE performed more than one year after myectomy demonstrated a significant increase in hyperemic myocardial perfusion in both the hypertrophied and non-hypertrophied segments in two subjects (Fig. 4), both of whom had severe LVOT obstruction at rest before myectomy. In these individuals, improvement in MBF post-myectomy was attributable primarily to an increase in microvascular flux rate. A decrease in stress perfusion post-myectomy was seen in the subject that had a high LVOT gradient only during exercise who also had very high exercise perfusion pre-myectomy.

## Discussion

Hypertrophic cardiomyopathy is a disease with a wide degree of phenotypic variability. Clinical studies demonstrating inducible ischemia in patients obstructive HCM were published four decades ago using non-quantitative radionuclide imaging [2, 22, 23]. Since then, quantitative perfusion imaging with positron emission tomography, CMR, and MCE in patients with HCM have confirmed that myocardial blood flow in the hypertrophied and non-hypertrophied regions is frequently reduced during exercise or vasodilator stress, and occasionally at rest [1, 3, 4, 7]. In the current study, we demonstrated that vasodilator stress MCE with regadenoson can be used to identify abnormalities in perfusion at rest and during stress in patients with HCM, and that the spatial manifestations of perfusion defects are varied, with subendocardial or patchy abnormalities being more common than transmural diffuse defects in hypertrophied segments. Substantial reduction in hyperemic perfusion during vasodilator stress is commonly found in those with large amounts of fibrosis detected by LGE. We also demonstrated that improvement in hyperemic perfusion in both the hypertrophied and non-hypertrophied regions can occur late after septal myectomy.

In HCM, reduced perfusion reserve in the absence of atherosclerotic CAD has been attributed, in part, to structural abnormalities of the vasculature. On histopathology, medial hyperplasia and lumen narrowing of small coronary arteries and arterioles, and a reduction in myocardial capillary density in hypertrophic regions has been described in HCM [8–10]. The latter feature indicates a failure in compensatory remodeling of the distal circulation to address the increased LV mass, cellular hypertrophy, and increased LV work and wall stress in HCM. Because of the compensatory reserve in the capacity of arterioles to dilate and capillary units to recruit, resting perfusion can be preserved in most patients with HCM. Yet, partial exhaustion of reserve and increased resistance from arteriolar narrowing and reduced capillary density is expected to produce myocardial ischemia during hyperemic stress or increased metabolic demand.

Functional abnormalities of the microcirculation in HCM have also been described. Extravascular compressive forces from high LV systolic and diastolic pressures in combination with the normal transmural pressure drop would be expected to reduce maximal flow in HCM, particularly in the endocardium. This mechanism has been proposed to explain reduced endocardial flow reserve in HCM, particularly in patients with high LV end-diastolic pressures or extreme septal hypertrophy [4, 7]. Abnormalities in the phasic flow of coronary arteries can occur from altered hemodynamic forces in HCM. Studies using invasive coronary flow wires or non-invasive coronary wave intensity measurements have revealed a marked predominance of diastolic flow and more prominent retrograde systolic flow in distal coronary arteries in subjects with HCM, which can be further accentuated by inotropic stress [12, 24]. Exaggerated retrograde flow combined with delayed or shortened diastolic relaxation can result in reduced antegrade discharge from small arteries or large arterioles that normally act as a type of “hydraulic capacitor” [12, 25]. From a clinical perspective, this functional abnormality is likely to worsen as LV end-systolic pressure, myocardial diastolic pressure, and heart rate increase.

In the current study, the spatial distribution of perfusion abnormalities during vasodilator stress, whether from structural or functional causes, was assessed by MCE. This technique provides parametric information on whether abnormalities in perfusion are secondary to reduced MBV, which can occur from either capillary rarefaction or functional non-patency of microvascular units [26]. It also measures microvascular flux rate which can be reduced from high resistance anywhere along the vascular network [27]. At rest, MCE revealed very modest reductions in MBV in both the hypertrophic and non-hypertrophic regions of patients with HCM despite these subjects having higher systolic wall stress and work. There is reason to believe that this abnormality was from abnormalities in phasic flow based on results from previous studies showing a high degree of cyclic video intensity at the LV apex, primarily from low systolic intensity, during resting MCE in patients with apical HCM [28]. Ordinarily, our finding of reduced perfusion at rest and increased work would be expected to result in ischemia. Yet these subjects were not symptomatic and LV systolic function was normal. This paradox could be related to compensatory mechanisms to increase oxygen delivery, even out of proportion to calculated work, based on studies using ^11^C-acetate PET indicating that myocardial oxygen consumption is not reduced in subjects with HCM who have normal to high LVEF [29].

Perfusion abnormalities in those with HCM became much more prominent during vasodilator stress, primarily because of a deficit in the ability to appropriately augment microvascular flux rate. This finding is somewhat different from previous quantitative MCE studies that found that reduced MBF in HCM, both at rest and during stress, is attributable to abnormal MBV [7]. We believe differences between the two studies can be explained by much less severe LVOT obstruction in the current study. We found that the dominant spatial pattern for hyperemic flow deficits was subendocardial or patchy in distribution. These patterns do not indicate any one mechanism since they could occur from pre-capillary drop in resistance from arteriolar narrowing, microvascular rarefaction, or phasic functional abnormalities of coronary flow. We observed a marked improvement in hyperemic flow, including in non-hypertrophied territories, after septal myectomy in two subjects who had very high resting LVOT gradients and low hyperemic flow (approximately one-third of control subject average) prior to surgical intervention. This finding suggests that abnormal flow from high systolic compressive forces in combination with delayed relaxation can affect global myocardial perfusion and is reversible late after correction of the high systolic gradient.

There are several important limitations of the study. The total number of subjects studied and the number of subjects undergoing myectomy was low because of strict entry criteria, including the need for recent CMR and exclusion for treatment with a myosin inhibitor which was being investigated concurrently with recruitment for this study. Yet data indicating a potential beneficial effect of myectomy on perfusion can be used to justify a larger prospective study in that narrow population of patients. Although MCE can be used to calculate absolute MBF in mL/min/g, this analysis was not performed because the requisite calculation of absolute MBV is valid only if blood pool microbubble signal is below the upper limit of the dynamic range which generally requires lower contrast infusion rates and appropriate scaling. Perfusion data were also not expressed as MBF normalized to work because of limitations in using end-systolic pressures to reflect total systolic load. Instead, we simply concluded that perfusion deficits in HCM occurred despite greater workload based on high systolic LV pressures. It should also be noted that vasodilator stress rather than exercise stress was used. The latter would provide a better test for stress-induced deficits in MBV, although the level of stress induced would be difficult based on difficulties in determining true afterload in those with dynamic gradients. Similarly, we have not tested other vasodilator agents, such as NO donors that could produce different results based on their prominent effects on pre-load which would affect wall stress and myocardial work, and based differences in the circulatory network where NO acts. Finally, ischemia from CAD was excluded by angiography in only about half of the HCM subjects, all of whom had anginal symptoms. One patient was excluded from analysis based on the presence of severe CAD on angiography performed for a typical coronary distribution of perfusion deficits on stress MCE.

## Conclusions

In summary, using vasodilator stress MCE we have demonstrated that hyperemic perfusion defects in HCM are common and manifest as a variety of different spatial patterns that vary according to whether they are present in the hypertrophied versus non-hypertrophied segments. Resting defects were primarily from abnormalities in functional MBV, whereas stress-induced defects were primarily from abnormalities in microvascular flux rate. Consistent with some other studies, there was not a strong relation between LVOT gradients and flow deficits. Yet those with the highest LVOT gradients all tended to have low hyperemic flow which is potentially treatable by myectomy.

## Supplementary Information

**Additional file 1:** **Supplemental Figure 1.** Illustration of the protocol used for quantitative myocardial contrast echocardiography. Contrast-specific imaging with pulse-inversion power-modulation imaging was used to suppress myocardial signal (dark) relative to microbubble signal (white), using a 55 dB dynamic range. A high-power (mechanical index) pulse was used to destroy microbubbles within the acoustic beam, thereby rendering the myocardium void of signal (dark). Over time, microbubble replenishment within the acoustic sector (apical 4-chamber plane in this example) occurs, illustrated by recovery of the myocardial contrast intensity in the end-systolic frames (top images) which correspond to microvascular refill (schematically illustrated in the middle panels). In the example shown, the heart rate was 60 bpm so that sequential end-systolic occur on a per-second basis. Time-intensity are then fit to a 1-exp function whereby the plateau intensity (A-value) after full replenishment represents the relative blood volume; and the rate constant (β) represents blood flux rate.

## Data Availability

The datasets used and/or analysed during the current study are available from the corresponding author on reasonable request.

## Abbreviations

CAD: Coronary artery disease
CMR: Cardiac magnetic resonance
HCM: Hypertrophic cardiomyopathy
ICD: Implantable cardioverter defibrillator
LGE: Late gadolinium enhancement
LV: Left ventricle
LVEF: Left ventricular ejection fraction
LVOT: Left ventricular outflow tract
MBF: Microvascular blood flow
MBV: Microvascular blood volume
MCE: Myocardial contrast echocardiography
SCD: Sudden cardiac death
