# Supplementary figures and images for: Myocardial contrast echocardiography assessment of perfusion abnormalities in hypertrophic cardiomyopathy
Source: Cardiovasc Ultrasound. 2022 Sep 19;20:23. doi: 10.1186/s12947-022-00293-2 (PMC9484161; doi:10.1186/s12947-022-00293-2)

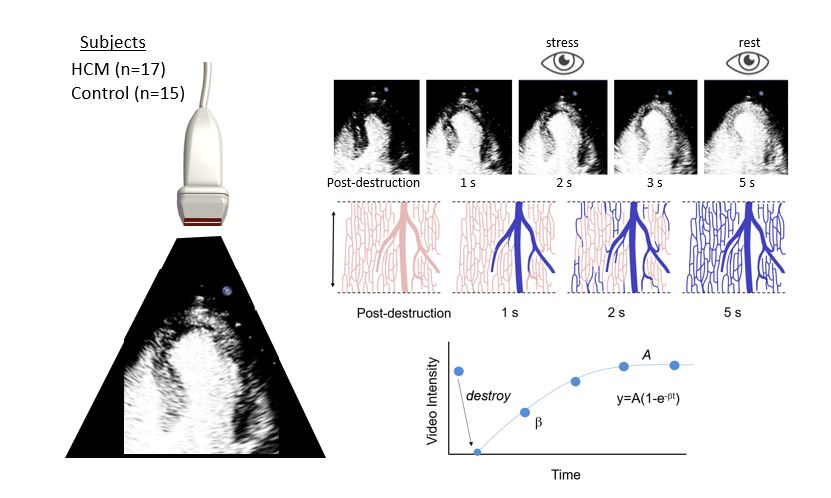

Supplement: Supplementary file 1 — Additional file 1: Supplemental Figure 1. Illustration of the protocol used for quantitative myocardial contrast echocardiography. Contrast-specific imaging with pulse-inversion power-modulation imaging was used to suppress myocardial signal (dark) relative to microbubble signal (white), using a 55 dB dynamic range. A high-power (mechanical index) pulse was used to destroy microbubbles within the acoustic beam, thereby rendering the myocardium void of signal (dark). Over time, microbubble replenishment within the acoustic sector (apical 4-chamber plane in this example) occurs, illustrated by recovery of the myocardial contrast intensity in the end-systolic frames (top images) which correspond to microvascular refill (schematically illustrated in the middle panels). In the example shown, the heart rate was 60 bpm so that sequential end-systolic occur on a per-second basis. Time-intensity are then fit to a 1-exp function whereby the plateau intensity (A-value) after full replenishment represents the relative blood volume; and the rate constant (β) represents blood flux rate. [file 12947_2022_293_MOESM1_ESM.jpg]
